# Agricultural intensification and climate change have increased the threat from weeds

**DOI:** 10.1111/gcb.15585

**Published:** 2021-03-23

**Authors:** Jonathan Storkey, Andrew Mead, John Addy, Andrew J. MacDonald

**Affiliations:** ^1^ Rothamsted Research Hertfordshire UK; ^2^ The Francis Crick Institute London UK

**Keywords:** climate change, crop yield, herbicides, integrated weed management, weed competition

## Abstract

Weeds represent a significant threat to crop yields and global food security. We analysed data on weed competition from the world's longest running agricultural experiment to ask whether potential yield losses from weeds have increased in response to management and environmental change since the advent of the Green Revolution in the 1960s. On plots where inorganic nitrogen fertiliser has been applied, potential yield losses from weeds have consistently increased since 1969. This was explained by a warming climate, measured as air temperature averaged over the growing season for the weeds, and a shift towards shorter crop cultivars. Weeds also reduced yield proportionally more on plots with higher rates of nitrogen which had higher yields when weeds were controlled; the relative benefit of herbicides was, therefore, proportional to potential crop yield. Reducing yield losses from weed competition is increasingly challenging because of the evolution of herbicide resistance. Our results demonstrate that weeds now represent a greater inherent threat to crop production than before the advent of herbicides and integrated, sustainable solutions to weed management are urgently needed to protect the high yield potential of modern crop genotypes.

## Graphical abstract

The world's longest running agricultural field experiment has plots that have never received herbicides. When yields from these plots were compared to those from equivalent plots where weeds are controlled with herbicides, we observed increasing yield losses between 1969 and 2014. This was explained by rising temperatures and the shift towards shorter crop cultivars.

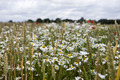

## 1 INTRODUCTION

**FIGURE 1:**
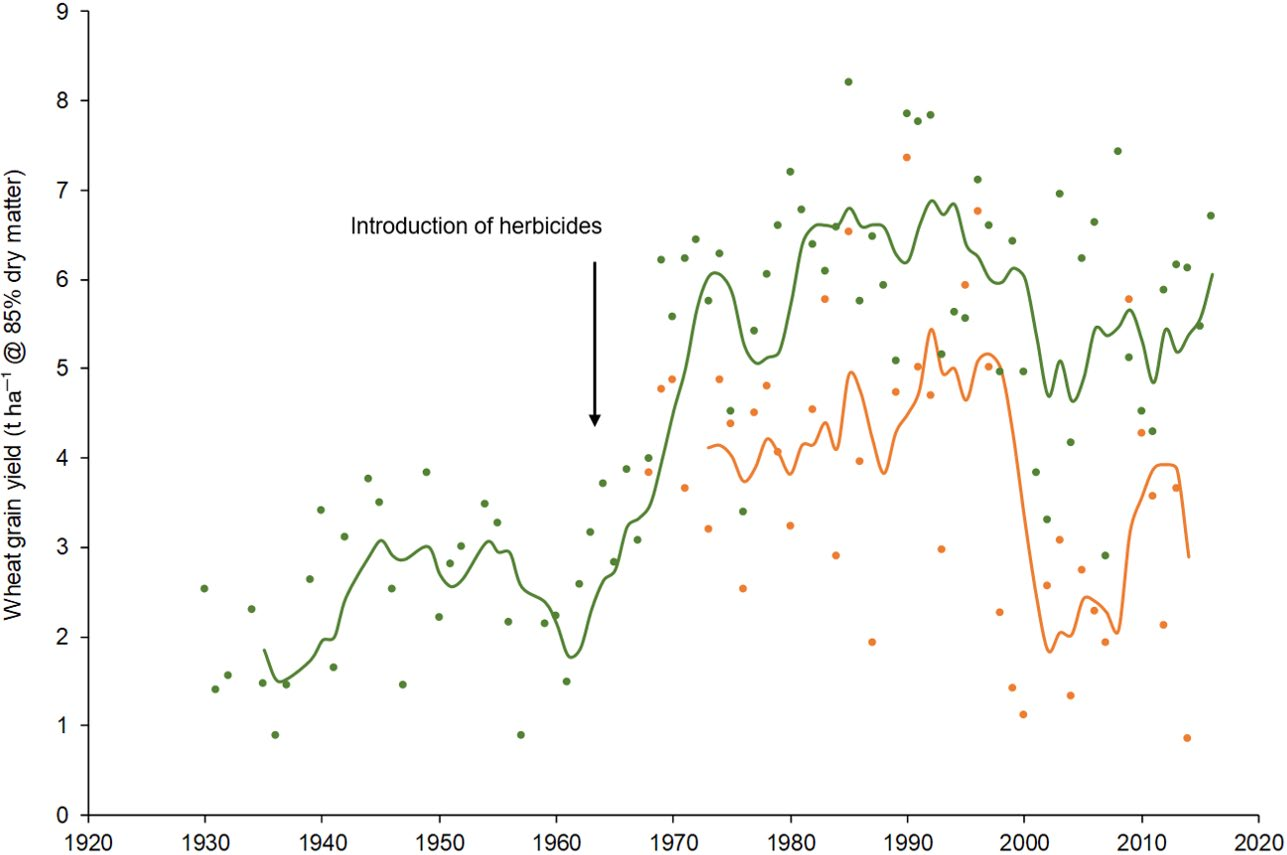
Historical yield trends on the Broadbalk experiment between 1930 and 2014, comparing two plots in a strip that have received a constant rate of nitrogen (144 kg N ha^−1^) since the start of the experiment in 1843 along with P, K and Mg. Data are presented for the plot in Section 8 that has never received herbicides (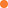) compared to the plot in Section 9 that has had herbicides applied since 1968 (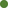). Before this date, weeds were controlled on both plots by hand weeding. Lines are moving 5‐year averages

The dramatic increase in crop productivity since the 1960s associated with the Green Revolution has delivered plentiful, cheap food for much of the world's expanding population (Pingali, 2012). These gains have largely been won through the enhanced yield potential of modern crop cultivars combined with the increased use of inorganic fertilisers (Borlaug, 2007; Evenson & Gollin, 2003). However, this increased yield potential is only realised in the context of effective chemical crop protection that limits yield losses from pests, weeds and diseases (Oerke, 2006). The importance of crop protection to food production is clearly demonstrated in data from the world's longest running field experiment, the Broadbalk winter wheat experiment at Rothamsted Research, Harpenden, UK which celebrated its 175th year in 2018 (Figure S1; Figure 1).

The Broadbalk experiment was started in 1843 and has plots on which winter wheat has been grown continuously (except for occasional fallows between 1926 and 1968) with contrasting treatments of different amounts and combinations of inorganic fertilisers compared with farmyard manure (Storkey et al., 2016). The original fertiliser treatments, arranged in ‘Strips’, were divided into 10 ‘Sections’ with contrasting management in 1968. One Section (Section 8) has never received herbicides that started being used on the rest of the experiment in 1964. These plots now represent a unique historical record of the interaction of management and environmental change on weed communities and their impact on crop production along a soil fertility gradient. Spatial and temporal variation in the potential yield loss from weeds can be quantified by comparing plots in the herbicide‐free Section 8 with neighbouring plots in Section 9 that have equivalent fertiliser treatments but have herbicides applied (Figure S1). Dramatic increases in yields were observed on Broadbalk following the introduction of modern short‐strawed varieties in 1968 reflecting gains in productivity of major crops globally (van Ittersum et al., 2013). However, these yield benefits were not fully realised on plots where weeds were not controlled with herbicides—a large proportion of the yield gains effectively being ‘robbed’ by the weeds (Figure 1).

The widespread use and efficacy of herbicides has meant that the large potential yield losses from weeds observed on the Broadbalk experiment have been largely avoided in world agriculture since the introduction of chemical weed control in the 1960s (Oerke, 2006). Consequently, there is a lack of data on temporal trends in potential yield loss from weeds owing to management or environmental change. However, these data are particularly important at this juncture because of the widespread evolution of resistance to herbicides (Mortensen et al., 2012; Neve et al., 2009) leading to increasing yield losses from weeds in intensively managed cropping systems. Epidemics of herbicide‐resistant species are now becoming increasingly common including *Alopecurus myosuroides* Huds. in northwest Europe (Hicks et al., 2018), *Amaranthus palmeri* S. Watson in southern and central United States (Ward et al., 2013) and *Lolium rigidum* Gaudin in Australia (Owen et al., 2014). While herbicides have been the dominant driver of weed community dynamics in recent decades, it is likely that other changes in crop management (Baessler & Klotz, 2006; Fried et al., 2012; Storkey et al., 2012) and the environment (Peters et al., 2014) have also had an impact on weed floras and weed competition.

**FIGURE 2:**
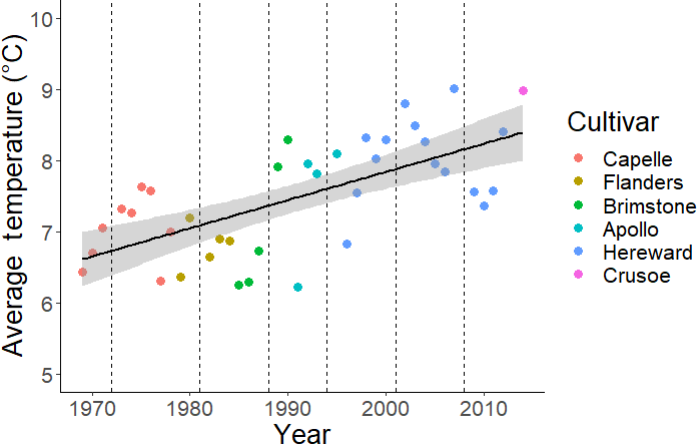
Environmental and management change on the Broadbalk experiment since 1969. Average temperature has been calculated for each year from the date of sowing to 30 June and changes in the wheat cultivar grown are indicated (Capelle‐Desprez has been shortened to Capelle) along with the dates of the fallows (‐‐‐). There has been a significant increase in average temperature over time: Average temperature =0.04 × year – 71.2 (*r* ^2^ = 0.42, *p *< 0.001).

In this context, the time series of yield data from Section 8 on Broadbalk is a valuable resource for identifying trends in weed competitiveness and their potential threat to food production. Any temporal trends in potential yield loss that are observed over the study period could be a consequence of either changes in management on the experiment, and/or environmental change. Management factors that would be expected to affect weed competitiveness include date of sowing, variation in the periodicity of fallows and changes in the cultivars being grown on the experiment; over the study period, Section 8 has been fallowed six times and six different cultivars have been grown on Broadbalk (Figure 2), sown between 28 September and 24 November. There have also been environmental trends recorded on the Rothamsted site over the study period. Between 1968 and 2014, in line with global trends (IPCC, 2014), average air temperatures measured at the meteorological site local to the Broadbalk experiment have risen consistently (Figure 2). Calculated over the main growing season for UK weeds (from crop sowing to 30 June), average temperatures have risen at a rate of 0.04°C year^−1^ and are now approximately 2°C higher than in 1969. There has been no significant trend in total precipitation over this period. Finally, although we expect weed populations to have plateaued and reached a dynamic equilibrium because of density‐dependent processes within a few years of weed control being removed (Freckleton & Watkinson, 2002; de Leon et al., 2014), the possibility of a long‐term trend in the overall weed burden on the plots also needs to be accounted for in the analysis. Accounting for all these factors, we answer the question of whether weeds now constitute a greater potential threat to yield than prior to the advent of herbicides.

## 2 METHODS

### 2.1 Description of Broadbalk data used in analysis

Six fertiliser strips were selected for the analysis representing a gradient of added inorganic nitrogen fertiliser (48–288 kg N ha^−1^; Table 1). Apart from a change in the nitrogen level for Strips 15 and 16, any other treatment changes have been applied uniformly across the six strips over the period analysed in the models (1969–2014). Specifically, Na and P stopped being applied in 1973 and 2000 respectively when they became supra‐optimal—for the purposes of this analysis, they can be assumed to be non‐limiting. Broadbalk yields are measured on a central 2.1 m wide strip in each plot harvested with a small plot combine, approximating to an area of 0.005 ha. Yield data recorded between 1969 and 2014 were extracted from the electronic Rothamsted Archive (e‐RA; Perryman et al., 2018) for plots in the selected strips from Section 8 (continuous wheat, no herbicides) and Section 9 (continuous wheat plus herbicides). When Section 8 yields are subtracted from the equivalent plots in Section 9, the differences represent the potential yield loss in t ha^−1^.

**TABLE 1:** Treatments on Broadbalk strips used in the analysis comprising an inorganic nitrogen series. P: 35 kg P ha^−1^ as triple superphosphate, (P): Phosphorus withheld, K: 90 kg K ha^−1^ as potassium sulphate, Mg: 12 kg Mg ha^−1^ as Kieserite (was 35 kg Mg ha^−1^ every third year 1974–2000, previously 11 kg Mg ha^−1^ as magnesium sulphate until 1973), (Na): 16 kg Na ha^−1^ as sodium sulphate until 1973, N as single application (mid‐April)—N1, N2, N3, N4, N5, N6: 48, 96, 144, 192, 240, 288 kg N ha^−1^ as ammonium nitrate since 1986, calcium ammonium nitrate 1968–1985, ammonium sulphate or sodium nitrate (N*) until 1967

Fertiliser strip	Treatments until 1967	Treatments from 1968	Treatments from 1985	Treatments from 2001
06	N1 P K Na Mg	N1 P K (Na) Mg	N1 P K Mg	N1 (P) K Mg
07	N2 P K Na Mg	N2 P K (Na) Mg	N2 P K Mg	N2 (P) K Mg
08	N3 P K Na Mg	N3 P K (Na) Mg	N3 P K Mg	N3 (P) K Mg
09	N4 P K Na Mg	N4 P K (Na) Mg	N4 P K Mg	N4 (P) K Mg
15	N2 P K Na Mg	N3 P K (Na) Mg	N5 P K Mg	N5 (P) K Mg
16	N*2 P K Na Mg	N2 P K (Na) Mg	N6 P K Mg	N6 (P) K Mg

Section 8 was fallowed six times during the time period analysed: in 1972, 1981, 1988, 1994, 2001 and 2008. In addition, in 2013 bad weather in the autumn meant the crop was not sown until the spring—this year was excluded from the analysis leaving a total of 38 individual years covering a period of 45 years. More recent yield data than 2014 were not included as Section 8 had a 2‐year fallow (for the first time in its history) in 2015–2016 meaning the subsequent yields could not be compared with those following a single‐year fallow. Despite cleaning of the grain by the plot combine, the yield data from Section 8 are still contaminated by weed seeds. However, data were available on the plots used in this study for 4 years (2011–2014) that compared combined yields to samples hand cleaned of weed seeds; there was a strong linear relationship (clean yield =1.004 × combine yield – 0.400, *r^2^* = 0.97, *p *< 0.001; Figure S2). This function was used to correct all combine yields from Section 8 included in the analyses.

On the Section 8 plot receiving low rates of nitrogen (Plot 6), there can be a beneficial effect of weeds on crop yield (facilitation) as leguminous species (*Medicago lupulina* L. and *Vicia sativa* L.), that are common on this plot, fix atmospheric nitrogen and facilitate crop growth. In contrast, on plots receiving high rates of nitrogen, weeds reduce yields in most years because of resource competition. To derive a continuous metric of the impact of weeds on crop yield that integrates facilitation and competition, the relative yield of weedy plots was calculated for each year using the data from Section 8 (weedy yield) and Section 9 (weed‐free yield):

Relativeweedyyield(RWY)=weedy yield/(weedy+weed‐free yield).

A value of RWY >0.5 is indicative of facilitation being the dominant process and RWY <0.5 of resource competition dominating biotic interactions with the crop.

### 2.2 Weed assessments

To analyse the effects of spatial and temporal changes in weed frequency and diversity on RWY, data from annual weed surveys were extracted from e‐RA. Standardised quantitative surveys only began in 1991; each year in June the presence/absence of all weed species is recorded in 25 random 0.1 m^2^ quadrats in all of the Section 8 plots. The resulting frequency of a species is not a direct measure of abundance (for which data are not available) but the relationship between the two is overwhelmingly positive (Warton & Hui, 2011) and, at this small scale, it is reasonable to assume that a more frequent species is more abundant. Excluding the years of fallow and the year with an atypical drilling date (2013), 20 years of data were available. Three derived variables were then calculated summarising the properties of the weed communities in each plot and year: (1) (ln) species richness (number of species recorded), (2) the geometric mean of species frequency (number of quadrats in which a species was recorded) and (3) Shannon diversity (Magurran, 2003) using species frequency data. One species, *Alopecurus myosuroides* Huds., was recorded in every quadrat in all plots and years and was effectively included as a constant in the calculation of these variables.

Between 1930 and 1979, additional survey data are available that used a nine‐point observational scale from ‘O’ (occasional) to ‘PPP’ (plentiful) assessed on a plot scale. The subjectivity of the categories means the data are of limited usefulness for elucidating temporal trends (Moss et al., 2004). However, the data were used to rank weed species in the period between 1969 and 1979 by converting the categories to an ordinal scale (1–9) and summing the scores across all years and plots for each species to allow a comparison with the more recent survey data.

### 2.3 Statistical analysis

Generalised linear mixed models (GLMMs) were used to explain spatial and temporal variation in RWY using a bionomial distribution with a Logit link function, estimated dispersion parameter and fitted using the method of Schall (1991) in the software Genstat (Payne, 1993). Models were built in stages and using data from three time periods: early (1969–1990), late (1991–2014) and the whole 45‐year study period. The early years covered the period in which the cultivars Capelle‐Desprez, Flanders and Brimstone were grown and the later years, Apollo, Hereward and Crusoe. For each time period, management variables were first included as fixed terms in the models (RWY ~ Cultivar + Added nitrogen + Years since last fallow + Sowdate [days after 1 September]). Year was then added to the models to test for an additional temporal trend once changes in management had been accounted for (…+Year). Finally, environmental and weed variables were added to the model before year to test whether they could explain any temporal trend (…Environmental|Weed variable + Year). Weed variables were only available for the late time period.

The Broadbalk experiment was established before the advent of modern statistical methods and lacks any replication or blocking structure. We could not, therefore, discount the effect of additional spatial variation between the plots in underlying soil properties or temporal effects of unmeasured changes in soil properties or the environment over the study period. Therefore, as well as being a fixed effect, Year was also included as a random term in the models, nested within fertiliser strip (Strip × Year). Within each dataset, models were compared using the Akaike information criterion (AIC) and the best model selected. Finally, the best model for the whole study period was run, dropping insignificant terms, but including interactions of cultivar with management and environmental variables. As Crusoe was only grown for 1 year in the study period, there were insufficient degrees of freedom to test interactions and this year (2014) was not included in the final model.

To analyse spatial and temporal trends in the relative frequency of weed species between 1991 and 2014, partial redundancy analysis (pRDA) was used; first analysing the effect of added nitrogen including year as a covariate and then including year as the explanatory variable with nitrogen as a covariate using the Canoco 5 software (Šmilauer & Lepš, 2014).

## 3 RESULTS

**FIGURE 3:**
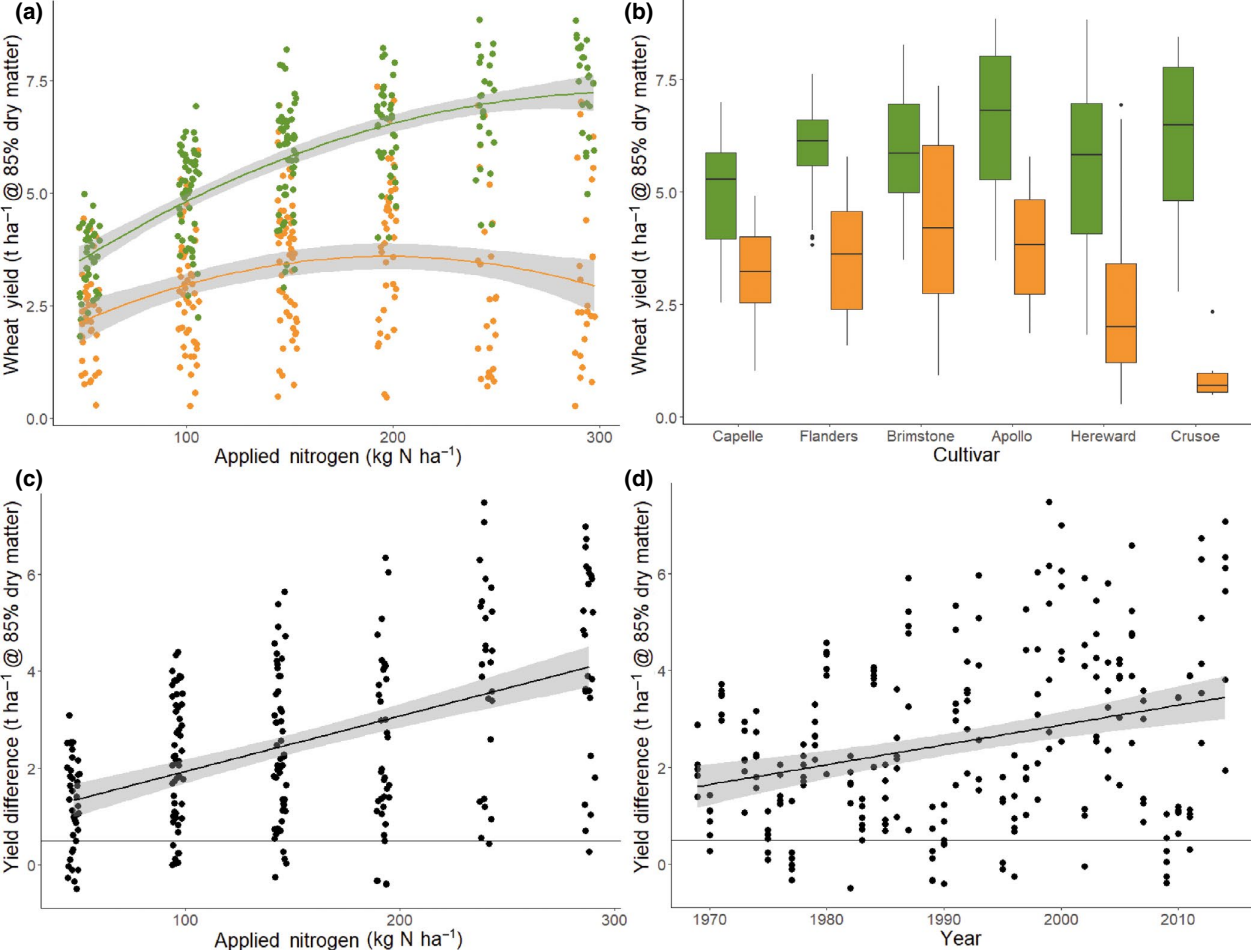
(a) Impact of increasing nitrogen fertiliser on wheat yields measured on plots with added nitrogen + herbicides (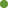) and with added nitrogen – herbicides (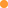); data have been jittered on the *x*‐axis. A quadratic function has been fitted to each dataset. (b) Wheat yield of cultivars (ordered sequentially) measured on plots with added nitrogen + herbicides (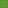) and with added nitrogen – herbicides (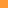). The lower and upper hinges correspond to the first and third quartiles (the 25th and 75th percentiles). The whiskers extend from the hinge to the largest/smallest value no further than 1.5 times the distance between the first and third quartiles. Outliers are plotted individually, (c, d) Potential yield loss expressed as the difference between weed‐free plots (+herbicides) and the weedy plots (−herbicides) from the same year and fertiliser strip plotted against nitrogen fertiliser (points jittered on *x*‐axis) and year. Both relationships are significant when modelled using least squares linear regression (*p* < 0.001)

When potential yield loss was expressed as the difference, in t ha^−1^, between the weed‐free and equivalent weedy plots in each fertiliser strip, there was an apparent spatial and temporal trend with yield losses increasing as more nitrogen was added and with time (Figure 3). As soil fertility increases, the negative impact of weeds on the Broadbalk experiment also increases (Figure 3c). Consequently, the classic asymptotic response curve of crop yield to increasing nitrogen fertiliser on the weed‐free plots is not observed on the weedy section (Figure 3a). Potential yield losses from weed competition also significantly increased with time since 1969 (Figure 3d) with an indication that this is partly explained by differences between the cultivars (Figure 3b). However, these spatial and temporal trends in yield loss, expressed as absolute t ha^−1^, may be a consequence of increases in the yield potential of more modern crop cultivars when grown with higher rates of nitrogen (Figure 3a,b). In addition, the temporal trend is also confounded by changes in the nitrogen treatments on some plots (Table 1) and variability in the length of time between fallows during the study period. These limitations in using absolute yield loss to interpret changes in the competitive balance between the crop and the weeds were overcome by building models to explain variance in proportional yield loss, expressed as RWY, and accounting for management changes before analysing the temporal trend.

When management variables were included in the GLMMs to explain variation in RWY, there was always a significant negative relationship with years since the last fallow and a positive relationship with sowing date (expressed as days since 1 September) for all time periods (Table 2; Figure S3). The effect of cultivar was also significant in all models. However, added nitrogen was not a significant term in the model for the early time period data. Adding year as a fixed factor was also non‐significant for the early time period and did not improve the model (the lowest AIC was attributed to the model that just included the management variables). However, there was a significant temporal trend in RWY, once management factors had been accounted for, when the data from 1991 to 2014 and the whole study period were analysed.

**TABLE 2:** Results of a generalised linear mixed model (*F* statistics) explaining variance in relative weedy yield (assuming a binomial distribution and using a logit link function) and including (plot × year) as random factors. The data are split into early (1968–1990) and late (1991–2014) periods before running the model on the whole dataset. The models were run first with just the management variables, then adding a temporal trend by including year as a fixed factor before also adding average temperature and precipitation (between sowing and 30 June) or, where data were available for the later period, the mean frequency of individual weed species, species richness and Shannon diversity calculated from presence/absence data recorded in twenty‐five 0.1 m^2^ quadrats in each plot and year. Model performance was compared using the Akaike information criterion (AIC) and the best model within each dataset is highlighted in bold

Additional variables in model	Management variables				Additional variable	Year	AIC
	Cultivar	Added nitrogen	Years since fallow	Sowing date			
1969–1990							
**None**	4.9^**^	**ns**	37.5^***^	40.3^***^	**—**	**—**	**77.0**
+Year	4.9^**^	ns	37.4^***^	40.2^***^	**—**	ns	81.3
+Temperature	5.2^**^	ns	38.9^***^	41.5^***^	ns	ns	80.7
+Precipitation	4.9^**^	ns	38.1^***^	40.9^***^	ns	ns	92.6
1991–2014							
None	10.9^***^	8.1^*^	105.6^***^	14.9^***^	**—**	**—**	162.4
+Year	17.8^***^	8.1^*^	103.3^***^	12.2^***^	**—**	5.7^*^	165.8
**+Temperature**	20.2^***^	8.4^*^	114.7^***^	13.1^***^	20.6^***^	**ns**	**157.4**
+Precipitation	20.2^***^	8.1^*^	116.0^***^	13.9^***^	25.2^***^	ns	165.4
+Weed richness	18.5^***^	7.5^*^	107.8^***^	12.6^***^	7.1^**^	4.9^*^	161.6
+Weed diversity	18.6^***^	6.7^*^	108.4^***^	12.6^***^	5.8^*^	6.3^*^	162.2
+Weed frequency	17.7^***^	8.0^*^	102.4^***^	12.1^***^	ns	5.3^*^	172.1
All years							
None	8.9^***^	5.4^*^	87.1^***^	25.0^***^	**—**	**—**	264.2
+Year	18.2^***^	5.4^*^	85.6^***^	21.9^***^	**—**	6.0^*^	267.8
**+Temperature**	19.3^**^	5.6^*^	91.1^***^	23.2^***^	23.2^***^	**ns**	**256.9**
+Precipitation	18.2^***^	5.4^*^	85.6^***^	21.9^***^	ns	4.6^*^	281.2

*, **, and *** indicate significance at levels of probability of *p* < 0.05, *p* < 0.01 and *p* < 0.001 respectively.

**FIGURE 4:**
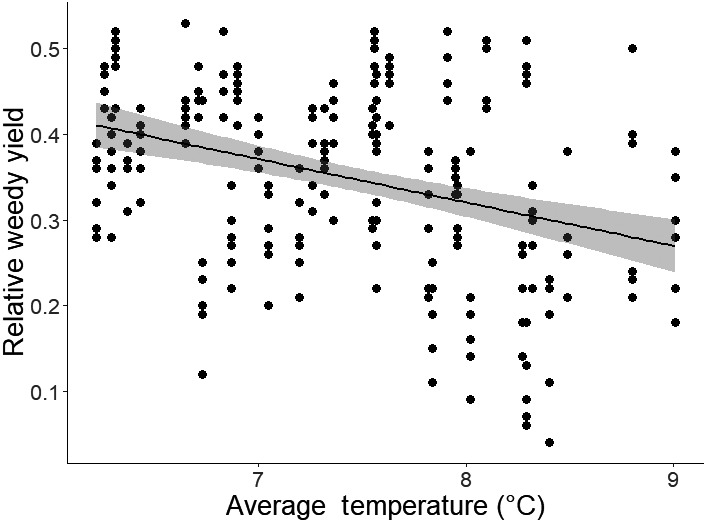
Relationship between relative weedy yield (RWY) and average temperature calculated between sowing and 30 June each year for the whole dataset (1969–2014). Temperature significantly explained variance in RWY after accounting for the effect of management change when combined in a generalised linear mixed model (*p* < 0.001) using a binomial distribution and logit link function

When average temperature over the growing season for weeds (sowing to 30 June) was included as an additional variable in the model, it significantly explained additional variance in RWY and year was no longer a significant term in the model. This was true both for the late time period and the whole study period (Figure 4) and implies that variation in mean temperature explains the observed temporal trend in RWY; these models also had the lowest AIC. For the late time period, a similar result was also observed for total precipitation calculated for the growing season of the weeds (Table 2). However, as the AIC indicated a worse fit of the model and precipitation was not a significant term in the model fitted to the whole dataset, this may be an artefact of the fact that total precipitation was significantly correlated with average temperature over the late time period (*r* = 0.59, *p *< 0.01).

The pRDA explaining temporal trends in the relative frequency of individual weed species between 1991 and 2014 (the period for which weed data were available) indicated a shift in community composition; three species had a significant negative response to year when nitrogen was included as a covariate, four increased and the remaining species had no significant trend (Figure S4b,c). There was a small net increase in mean frequency/species over time but there was no temporal trend in species richness or Shannon diversity. For six of the seven species which had a significant temporal trend in their frequency between 1991 and 2014, there was also an equivalent change in their ranking when compared to the earlier survey between 1969 and 1979, suggesting that a continuing trend was being observed in the later survey data. There were no clear relationships between species temporal trends and plant attributes (Table S1). The group of increasing species included different life forms and a range of Ellenberg indicator values for nitrogen and moisture. The analysis of the effect of nitrogen on weed communities (Figure S4a) confirmed the strong filtering effect of fertilisers on weeds, selecting for more nitrophilous, competitive species (Moss et al., 2004; Storkey et al., 2010). This is reflected in species with high Ellenberg N values being associated with plots with higher rates of nitrogen fertiliser (Table S1).

When the metrics of weediness were included in the models, year remained as a significant term indicating that any changes over time in the weed communities did not account for the temporal trend in RWY. However, it is notable that both weed richness and diversity explained additional variance in RWY; the impact of weeds on crop yield was lower than expected on plots with more diverse weed communities once the effects of treatment and management had been accounted for.

When the best model for the whole study period, retaining all management variables and average temperature, was subsequently run including interaction terms with cultivar, interactions between cultivars and fallowing, sowing date and average temperature were retained in the model but there was no interaction with applied nitrogen (Table 3). There was a disproportionate negative impact of time since fallow on RWY for the three more recent cultivars, Brimstone, Apollo and Hereward, when compared to Capelle‐Desprez and Flanders. The estimated RWY became progressively more negative for the recent cultivars in contrast with the older cultivars as the period since the last fallow lengthened. While the response of RWY to average temperature was again negative for four of the cultivars, Apollo had a positive response and was also less affected by sowing date. However, this result needs to be interpreted in the context of the relatively few years Apollo appears in the dataset (with limited ranges of explanatory variables; Figure 2).

**TABLE 3:** Estimates of model parameters, denominator degrees of freedom (ddf) and *F* statistics from a generalised linear mixed model explaining variance in relative weedy yield (RWY) including interactions of management and environmental variables with cultivar (RWY ~ cultivar × (Nitrogen + Fallow + Sowdate + Temperature)). There was no significant interaction of nitrogen with cultivar; only the parameter for the reference cultivar, Apollo, is shown. The cultivar Crusoe was only grown in a single year and has been excluded from the analysis

	Capelle‐Desprez	Flanders	Brimstone	Apollo	Hereward	ddf	*F* statistic
Intercept	−0.624	−0.761	−0.462	−0.571	−0.505	131	11.5^***^
Nitrogen				−0.0013		14	5.8^*^
Cultivar × Fallow	−0.010	−0.028	−0.320	−0.197	−0.311	194	35.9^***^
Cultivar × Sowdate	0.014	0.021	−0.004	−0.003	0.024	194	6.5^***^
Cultivar × Temperature	−0.061	−0.365	−0.279	0.163	−0.269	194	5.8^***^

*, **, and *** indicate significance at levels of probability of *p* < 0.05, *p* < 0.01 and *p* < 0.001 respectively.

## 4 DISCUSSION

**FIGURE 5:**
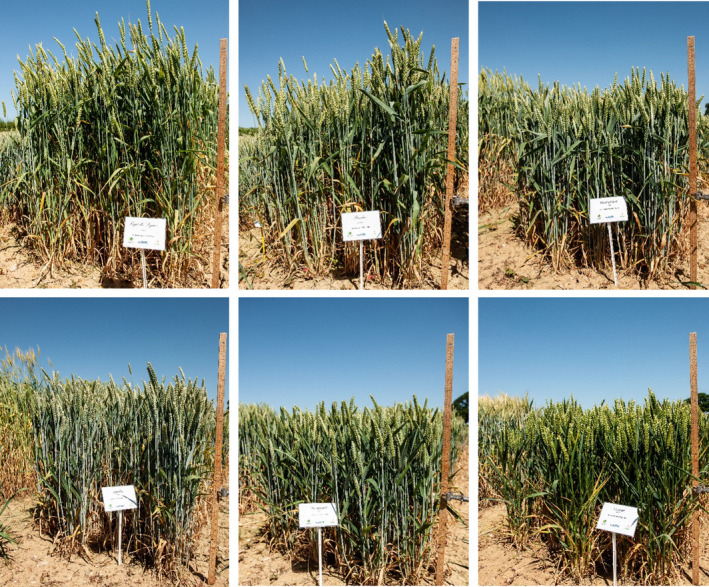
Comparison of cultivars grown on the Broadbalk experiment since 1968. From top left to bottom right: Capelle‐Desprez (1968–1978), Flanders (1979–1984), Brimstone (1985–1990), Apollo (1991–1995), Hereward (1996–2012), Crusoe (2013–2018). Meter rule included for scale

The analysis of spatial and temporal trends in weed competition on the Broadbalk experiment provides compelling evidence that the combination of management and environmental change since the start of the Green Revolution has increased the inherent threat from agricultural weeds. The response of RWY to changes in management and environment can be interpreted in terms of the shift in the competitive balance between the crop and the weeds. On the plots receiving nitrogen fertiliser, where the dominant process is resource competition, the decrease in RWY over time represents an increasing advantage to the weeds over the crop. The change in crop cultivars grown over the study period partly explained the observed increase in weed competition on these plots. The greater yield potential of modern crop varieties is largely associated with selection for dwarfing genes which decrease canopy height and increase harvest index (Evenson & Gollin, 2003). This is apparent in the time series of six wheat cultivars grown on the Broadbalk experiment over the period of our analysis (Figure 5). It is likely that the increasing relative difference in competitiveness between the cultivars with time since the last fallow is a result of decreasing crop height, reducing the ability of the crop to shade and suppress weeds (Andrew et al., 2015). As we move beyond an age that is overly reliant on chemical inputs and biotechnology for maintaining productivity (Mortensen et al., 2012), it is likely that plant breeders will need to consider traits that reduce yield losses from weeds as well as those that optimise yield potential in the absence of weed competition (Andrew et al., 2015).

Along with the introduction of high yielding, short crop cultivars, the intensification of agriculture has been characterised by increasing use of inorganic fertilisers. In this regard, Broadbalk represents a space for time substitution; the nitrogen gradient of increasing inputs and soil fertility providing an insight into the historical impact of intensification on weed competition. As nitrogen fertiliser rates increase, the weeds on Section 8 become progressively more competitive with a negative relationship between total nitrogen and RWY. This is largely a result of shifts in community composition along the soil fertility gradient from a more diverse, stress‐tolerant (and leguminous) weed community to less diverse communities dominated by nitrophilous, competitive species (Moss et al., 2004; Storkey et al., 2010), a trend that has been observed in the wider agricultural environment as fertiliser rates have increased (Storkey et al., 2012). The combined effect of modern cultivars and increasing nitrogen inputs on potential weed losses from weeds means that, in the absence of herbicides, only a fraction of the yield gains achieved through the Green Revolution are realised. Consequently, intensive cropping systems are now more reliant on effective weed control and the relative benefit of herbicides is proportional to potential crop yield. This phenomenon may partly explain the lack of a response of crop yield to herbicide use on farms with a range of potential yields (Gaba et al., 2016).

The negative temporal trend of RWY was not explained by any of the variables derived from weed frequency data. In addition, no temporal trend in RWY was observed for the early time period (1969–1990) before the increase in temperatures observed from 1991 onwards (Figure 2). This supports our hypothesis that any observed long‐term trend in RWY could not be explained by a continuous build‐up of weeds (that we would expect to be most pronounced in the earlier years) but, rather, density dependence mechanisms resulted in weed abundance reaching a dynamic equilibrium relatively quickly following the cessation of weed control. Although we might expect short‐term variation in weed seedling density as a result of fallowing and variable sowing dates, we conclude, therefore that the observed long‐term temporal trend in RWY is a result of environmental change that has shifted the competitive balance in favour of the weeds. This can be explained by the fact that weeds tend to compensate for a smaller seedling than the crop with a higher relative growth rate and are predicted to be more responsive to a warmer growing season than the crop (Storkey, 2004).

Since the advent of the Green revolution, large‐scale changes in weed diversity and species composition have been observed in several countries with shifts towards more nitrophilous, ruderal species and away from more stress‐tolerant communities (Fried et al., 2012; Meyer et al., 2013). These shifts can largely be attributed to changes in crop management (including increased fertiliser use). However, we would also expect climate change to impact the composition of weed floras as species differ in attributes such as tolerance to drought or germination periodicity. On Broadbalk, species that increased over the study period did not share similar plant attributes indicating that there had not been a consistent functional shift in the weed flora of this type. A previous study has quantified the relative importance of intra and interspecific competition in driving weed community dynamics on Broadbalk (de Leon et al., 2014). The authors identified seasonal temperature as a factor that shifted the competitive balance between species and identified functionally equivalent pairs of species that responded differently to temperature (a mechanism explaining coexistence). Species that were favoured by higher temperatures included *Vicia sativa* L. and *Ranunculus arvensis* L. Both these species also increased in frequency over the study period in our results. We conclude that temporal shifts in the composition of the weed flora are being driven by physiological differences in species’ response to the environment that determine the outcome of competitive interactions in the community. Finally, it is possible that variation in sowing date also selected between species based on variance in periodicity of germination; collecting data on the future on seedling densities on Broadbalk would address this knowledge gap.

In the context of the increasing competitiveness of weeds in response to intensification and climate change, our analysis provides support for the efficacy of two ‘cultural’ control options for weeds, fallowing and delayed drilling. Before the introduction of herbicides, fallowing represented a time‐honoured method of weed control (Derksen et al., 1994) and was first instituted on the Broadbalk experiment in 1926 when the experiment was divided into five sections. This was necessitated by the shortage of labour for hand weeding following the First World War. The ongoing efficacy of fallows on Section 8 is demonstrated by the negative relationship of RWY with years since fallow. For strips receiving nitrogen, this is likely to be a result of reduced weed abundance in the years immediately after a fallow. Delaying sowing date is also a well‐established strategy for reducing weed pressure as it reduces the proportion of a weed cohort emerging in the crop (Lutman et al., 2013) and reduces the competitive ability of the weeds (Andrew & Storkey, 2017); these effects were seen clearly in our data with a consistent positive relationship between sowing date and RWY.

As instances of herbicide resistance increase and the rate of introduction of new herbicides continues to slow, it has been suggested that herbicides represent a once in a century opportunity for weed control (Davis & Frisvold, 2017). While herbicides remain effective, underlying trends in the competitive dynamics of weeds and their potential threat to food production have been obscured. However, our results show that management and climate change have combined over the past 45 years to increase the threat from weeds. If we could no longer rely on herbicides, it could be argued that, in terms of weed pressure, the situation is now worse than before their widespread introduction in the 1960s. Our results, therefore, highlight the need to diversify weed control strategies by complementing herbicides with non‐chemical options including increasing crop competition and disrupting weed life cycles using fallows or more diverse cropping rotations. Only then will the high yield potential of modern crop genotypes be maintained.

## AUTHOR CONTRIBUTIONS

Jonathan Storkey conceived the ideas. All authors designed the analysis and analysed the data. Jonathan Storkey led the writing of the manuscript. All authors contributed to the final draft and gave final approval for publication.

## Supporting information

Supplementary Material

## Data Availability

The data used in this study are stored in the electronic Rothamsted Archive (e‐RA) online repository (http://www.era.rothamsted.ac.uk) and are available on request from the e‐RA curators (era@rothamsted.ac.uk).
