# Supplementary material for: Agricultural intensification and climate change have increased the threat from weeds
Source: Glob Chang Biol. 2021 Mar 23;27(11):2416–25. doi: 10.1111/gcb.15585 (PMC8252568; doi:10.1111/gcb.15585)
Supplement: Supplementary file 1 — Supplementary Material [file GCB-27-2416-s001.docx]

**Figure S1.** Current plan of the Broadbalk Experiment. Plots with the same fertiliser treatment are arranged in ‘Strips’ that were divided into ten ‘Sections’ in 1968. Continuous winter wheat is grown in five of these sections with the remaining five in a rotation with each rotational Section in a different phase. Section 8 has never received any herbicides which started being used on the rest of the experiment in 1964. Data from Sections 8 & 9 and Strips 6,7,8,9,15 & 16, that represent a gradient of inorganic Nitrogen fertiliser rates, were used in this study.


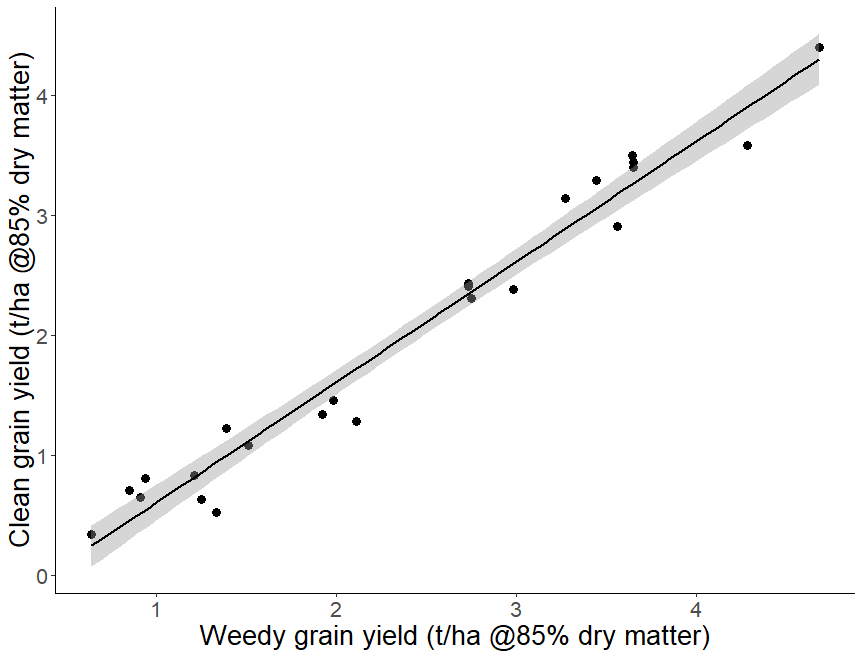


**Figure S2.** Relationship between yields from combine (contaminated with weed seed) and hand-cleaned grain for fertiliser strips 6, 7, 8, 9, 15 & 16 from Section 8 of the Broadbalk experiment for four years: 2011 - 2014. Cleaned grain = 1.004 * combine yield – 0.3998 (*r^2^* = 0.97, *P*<0.001).

| a) | b) |
| --- | --- |
| 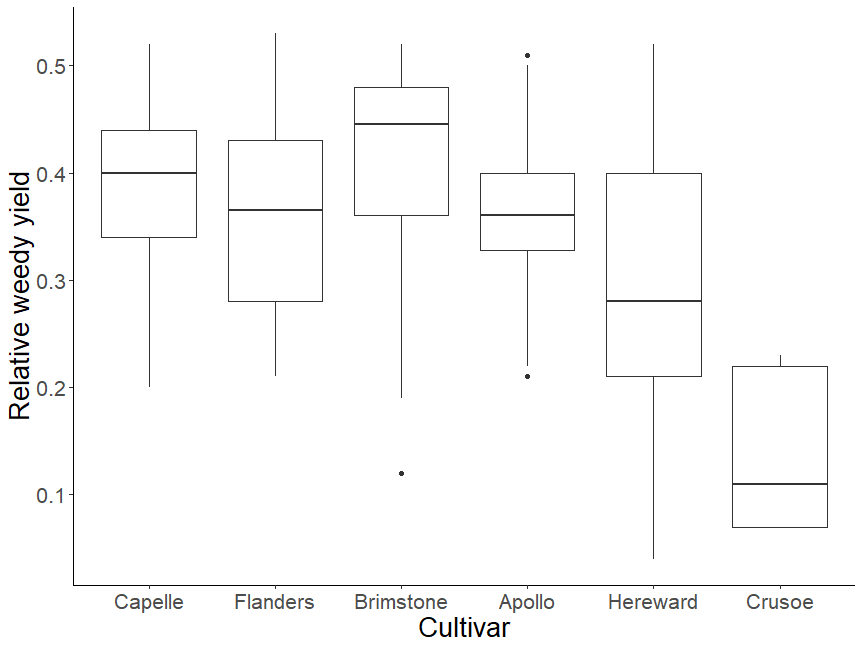 | 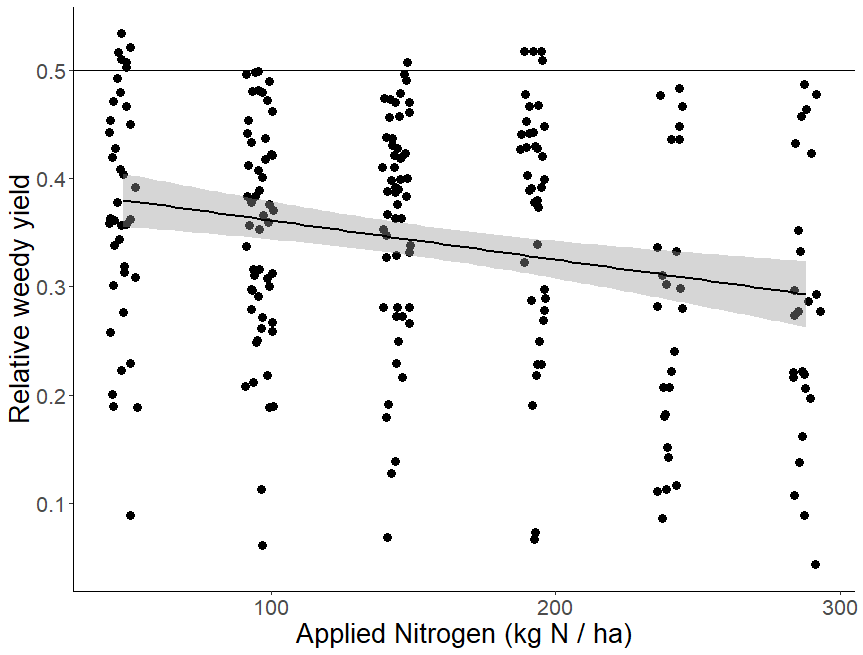 |
| c) | d) |
| 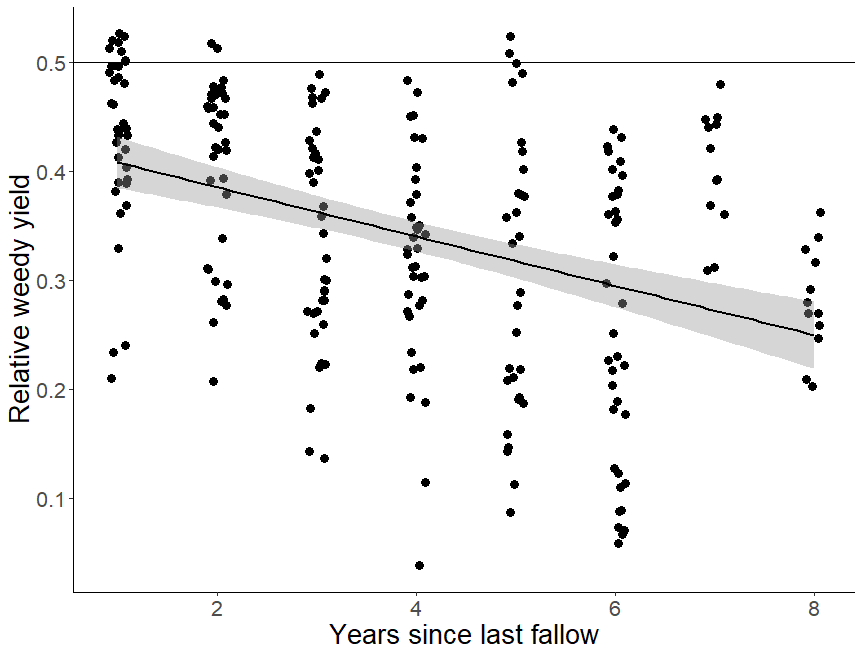 | 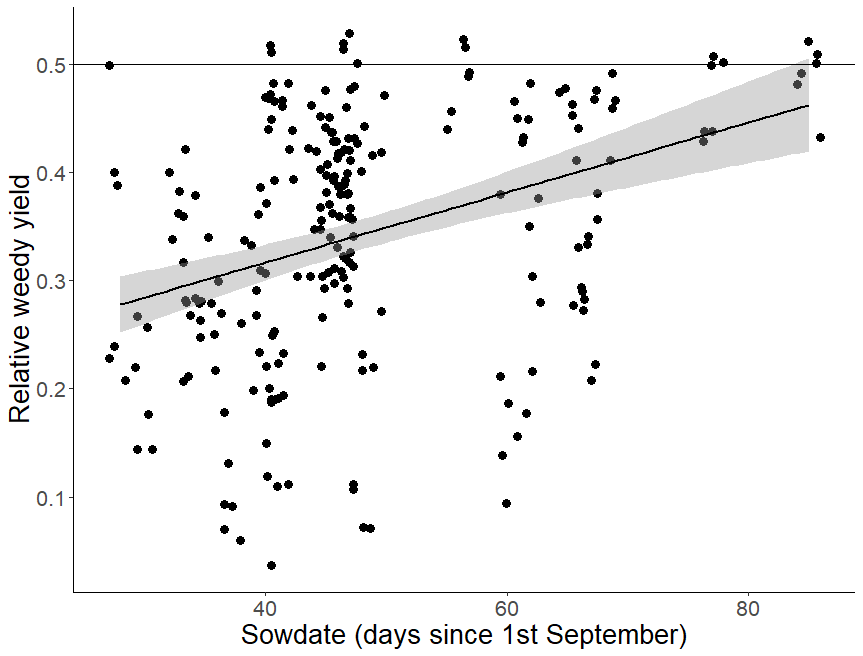 |

**Figure S3.** Relationship between Relative Weedy Yield and a) cultivars (Capelle-Desprez shortened to Capelle) arranged in chronological order b) applied Nitrogen, c) years since last fallow and d) sowing date of the crop expressed as days since 1^st^ September for whole dataset (1969-2014). All management variables significantly explained variance in RWY when combined in a GLMM (P<0.05) using a binomial distribution and logit link function.

a) b)


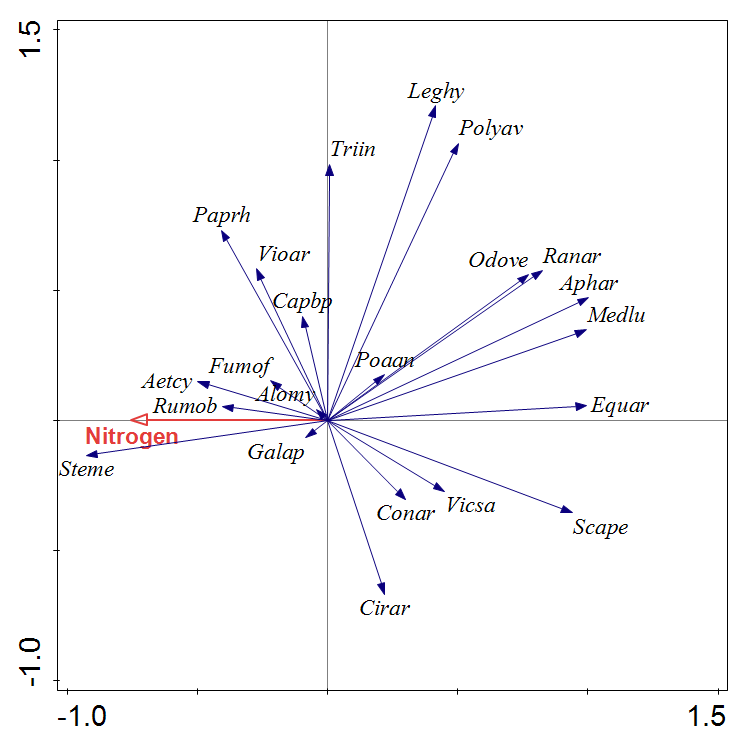

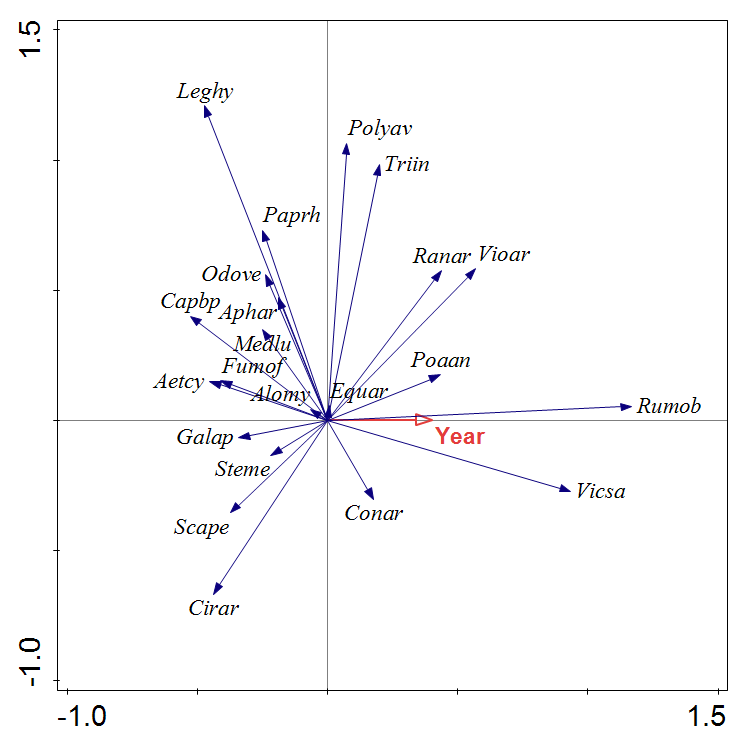


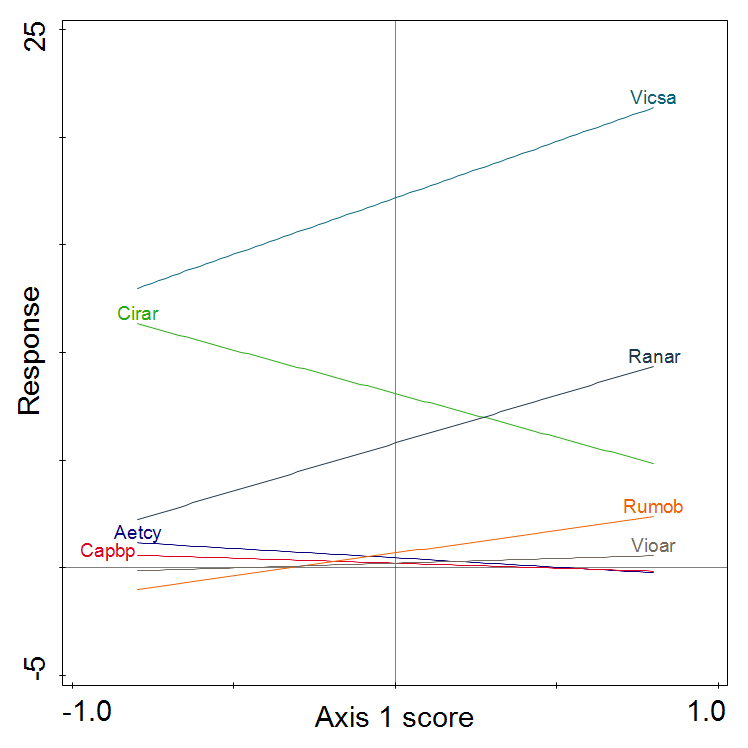
c)

**Figure S4.** Results of partial Redundancy Analysis (pRDA) of spatial and temporal trends in weed communities on Strips 6,7,8,9,15 & 16 of Section 8 (no herbicides) of the Broadbalk experiment recorded between 1991 and 2014: a) effect of increasing rate of inorganic Nitrogen fertiliser with Year as covariate (Nitrogen accounts for 33% variance, *P=*0.001), b) effect of Year with Nitrogen fertiliser as covariate (Year accounts for 4% variance, *P*=0.001), c) species responses to Year (using a GLM with identity link) in the pRDA with Nitrogen as covariate, plotted against Axis 1 scores; only species with a significant response are included (*P<*0.05). Species are labelled with first three letters of Genus and first two letters of Species (Table S1).

| Species | Rank in later survey (compared to earlier survey)* | Ellenberg  Nitrogen | Ellenberg Moisture | Raunkier  Life form | Status | Recent trend |
| --- | --- | --- | --- | --- | --- | --- |
| *Alopecurus myosuroides* Huds. | 1 (1) | 6 | 5 | Therophyte | Archaeophyte |  |
| *Vicia sativa* L. | 2 (3) | 4 | 4 | Therophyte | Native | 🡩 (P=0.002) |
| *Aphanes arvensis* L. | 3 (7) | 4 | 4 | Therophyte | Native |  |
| *Papaver rhoeas* L. | 4 (4) | 6 | 5 | Therophyte | Archaeophyte |  |
| *Scandix pecten-veneris* L. | 5 (6) | 4 | 4 | Therophyte | Archaeophyte |  |
| *Tripleurospermum inodorum* (L.) Sch. Bip. | 6 (9) | 6 | 5 | Therophyte | Archaeophyte |  |
| *Cirsium arvense* (L.) Scop. | 7 (2) | 6 | 6 | Non-bulbous geophyte | Native | 🡫 (P=0.006) |
| *Legousia hybrida* (L.) Delarbre | 8 (14.5) | 4 | 4 | Therophyte | Archaeophyte |  |
| *Ranunculus arvensis* L. | 9 (5) | 6 | 5 | Therophyte | Archaeophyte | 🡩 (P=0.009) |
| *Stellaria media* (L.) Vill. | 10 (10) | 7 | 5 | Therophyte | Native |  |
| *Medicago lupulina* L. | 11 (13) | 4 | 4 | Therophyte | Native |  |
| *Polygonum aviculare* L. | 12 (8) | 7 | 5 | Therophyte | Native |  |
| *Equisetum arvense* L. | 13 (12) | 6 | 6 | Non-bulbous geophyte | Native |  |
| *Odontites vernus* (Bellardi) Dumort | 14 (18) | 5 | 5 | Therophyte | Native |  |
| *Rumex obtusifolius* L. | 15 (29) | 9 | 5 | Hemicryptophyte | Native | 🡩 (P<0.001) |
| *Poa annua* L. | 16 (23) | 7 | 5 | Therophyte | Native |  |
| *Capsella bursa-pastoris* (L.) Medik. | 17 (14.5) | 7 | 5 | Therophyte | Archaeophyte | 🡫 (P=0.013) |
| *Viola arvensis* Murray | 18 (29) | 6 | 4 | Therophyte | Archaeophyte | 🡩 (P=0.008) |
| *Convolvulus arvensis* L. | 19.5 (29) | 6 | 4 | Non-bulbous geophyte | Native |  |
| *Galium aparine* L. | 19.5 (11) | 8 | 6 | Therophyte | Native |  |
| *Fumaria officinalis* L. | 21 (17) | 6 | 5 | Therophyte | Archaeophyte |  |
| *Papaver argemone* L. | 22.5 (29) | 5 | 4 | Therophyte | Archaeophyte |  |
| *Valerianella dentata* (L.) Pollich | 22.5 (29) | 4 | 4 | Therophyte | Archaeophyte |  |
| *Aethusa cynapium* L. | 25.5 (16) | 6 | 4 | Therophyte | Archaeophyte | 🡫 (P=0.045) |
| *Chenopodium album* L. | 25.5 (29) | 7 | 5 | Therophyte | Native |  |
| *Elytrigia repens* (L.) Desv. ex Nevski | 25.5 (29) | 7 | 5 | Hemicryptophyte | Native |  |
| *Lamium purpureum* L. | 25.5 (29) | 7 | 5 | Therophyte | Archaeophyte |  |
| *Fallopia convolvulus* L. | 28 20.5) | 5 | 4 | Therophyte | Archaeophyte |  |
| *Arenaria serpyllifolia* L. | 29.5 (29) | 5 | 3 | Therophyte | Native |  |
| *Myosotis arvensis* (L.) Hill | 29.5 (20.5) | 6 | 5 | Therophyte | Archaeophyte |  |
| *Anagallis arvensis* L. | 32 (29) | 5 | 4 | Therophyte | Native |  |
| *Atriplex patula* L. | 32 (20.5) | 7 | 5 | Therophyte | Native |  |
| *Avena fatua* L. | 32 (29) | 7 | 4 | Therophyte | Archaeophyte |  |
| *Senecio jacobaea* L. | 34 (20.5) | 4 | 4 | Hemicryptophyte | Native |  |

*Using only data from Strips 6,7,8 & 9 for which fertiliser treatments remained constant between the two surveys

**Table S1.** Full species list of weeds recorded on Strips 6,7,8,9,15 & 16 on Section 8 (no herbicides) of the Broadbalk experiment between 1969 and 2014 using nomenclature of Stace (1997). Species have been arranged in rank order (most frequent first) based on the total number of quadrats in which a species was recorded across all years of the later survey (1991-2014). This is compared to the rank in the earlier survey (1969-1979) based on the observational scores on an ordinal scale for each species summed across Strips and years. Species attributes are derived from (Hill, Preston, & Roy, 2004). The trend of species that were identified as having a significant response to Year in the pRDA using the data from 1991-2014 is also indicated.

**References**

Hill, M. O., Preston, C. D., & Roy, D. B. (2004). *Planatt: Attributes of British and Irish Plants, Status, Size, Life History, Geography and Habitats.* Cambridgeshire, UK: Centre of Ecology & Hydrology.

Stace, C. A. (1997). *New Flora of the British Isles* (2nd ed.): Cambridge University Press.
